# In Situ Synthesis of Keratin and Melanin Chromophoric Submicron Particles

**DOI:** 10.1021/acsomega.3c00189

**Published:** 2023-07-05

**Authors:** Chen Nowogrodski, Yaniv Damatov, Sunaina Sapru, Oded Shoseyov

**Affiliations:** Plant Molecular Biology and Nano Biotechnology, Faculty of Agriculture, Food and Environment, The Hebrew University of Jerusalem, Rehovot 7610001, Israel

## Abstract

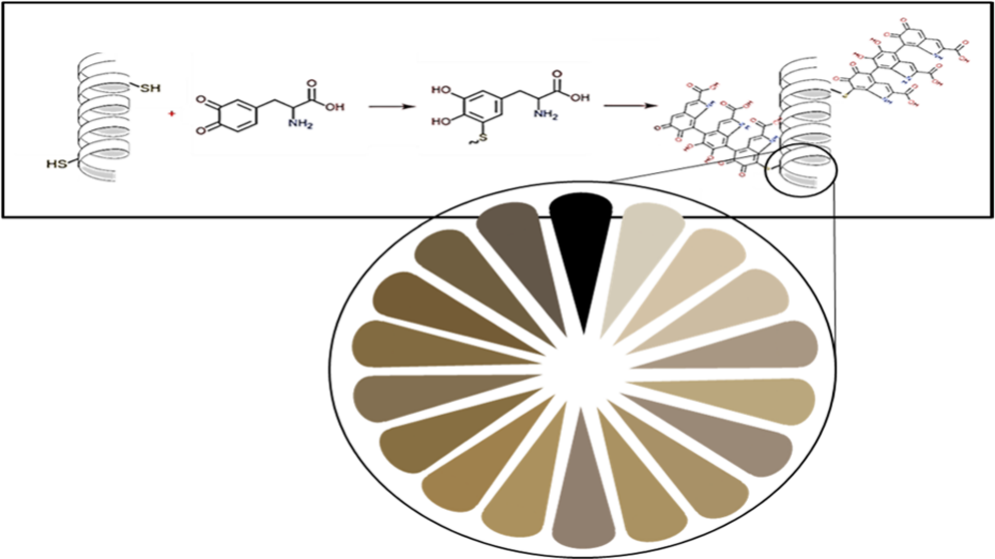

In humans, melanin plays an esthetic role, dictating hair and skin color and traits, while keratin is the protein that comprises most of the epidermis layer. Eumelanin and pheomelanin are types of melanin synthesized from the same building blocks via enzymatic oxidation. Pheomelanin has an additional building block, cysteine amino acid, which affects its final structure. Keratin contains high cysteine content, and by exploiting free thiols in hydrolyzed keratin, we demonstrate the formation of keratin–melanin (KerMel) chromophoric submicron particles. Cryo-TEM analyses found KerMel particle sizes to be 100–300 nm and arranged in the form of a central keratin particle with polymerized l-dopa chains. Attenuated total reflection (ATR)-FTIR, UV–vis, and fluorescence measurements identified new chemical bonds, indicating the formation of KerMel particles. Finally, KerMel replicated natural skin tones and proved cytocompatibility for human epidermal keratinocytes at concentrations below 0.1 mg/mL. Taken together, KerMel is a novel, tunable material that has the potential to integrate into the cosmetic industry.

## Introduction

The interest in biomimicry has increased in the past few years due to its potential to exploit the unique properties of biomaterial, especially for human skin replacement.^1,2^ When pursuing human skin, the substitute must closely resemble the natural tissue and exhibit integrity and pigmentation. Keratin is an ortholog protein in mammals and is the main component of the epidermis, serving as a scaffold for epithelial cells to withstand mechanical stress.^3^ Its unique physicochemical characteristics have brought it to the focus of investigation in various fields, such as tissue engineering and food packaging.^4−9^ Keratin is roughly divided into soft and hard keratins; the latter features a high sulfur content and a nanometric α helix main structure. The structural integrity of keratin assures by disulfide, hydrophobic, ionic, and hydrophobic bonds.^10,11^ Its secondary structure features various functional groups, which can chemically and physically react with other materials.^12−14^ Regenerated keratin relies on renewed disulfide bonds originating in the cysteine side chains when the thiol is a Michael acceptor with a strong nucleophilic side.^13,15^ Constant attempts are to improve regenerative keratin properties, such as thermal and mechanical properties, using various methods such as additives, plasticizers, and graft polymerization.^5,6,13^ Melanin is a ubiquitous material found in mammals, birds, and insects. In humans, melanin is synthesized by melanocytes and acts like a parasol, protecting the keratinocyte DNA from UV damage at the epidermis layer of the skin. Melanocytes extract melanin, the primary determinant of skin and hair pigmentation.^16^ Melanin synthesis, or melanogenesis, is described in the “Raper–Mason” scheme, where the pigment is synthesized from the oxidation of tyrosine to dopaquinone via tyrosinase (TYR).^17^ The mechanism of eumelanin and pheomelanin differs in the synthesis pathway.^18−21^ Oxidation of dopaquinone leads to the polymerization of eumelanin, a brown-black pigment. When cysteine is available, cysteinyldopa intermediates are formed, further polymerizing to pheomelanin, a yellow-red pigment. Both pigments are amorphic and tend to create stacked nanometric oligomers. Pigment expression depends on the concentration and ratio between eumelanin and pheomelanin. Both are heterogeneous polymers with inherent diversity and complexity, several monomers and polymerization variations, and an indirect link to genetics;^22^ therefore, their final structures are still being debated. Tyrosine and its derivatives, such as l-dopa, have been used to synthesize synthetic melanin. They can quickly oxidize and react with functional groups such as amine and thiol via Michael-type/Schiff base reactions.^23−26^ In addition, successful attempts to achieve synthetic pigments from the melanin pathway have relied on their polymerization in tyrosine-containing peptides or on polyethylene nanoparticles.^27,28^ Several works have focused on combining keratin and melanin building blocks and their derivatives, such as dopamine, polydopamine (PDA), and l-dopa. One group developed PDA-coated keratin films for improved photoactivity and keratin–PDA hydrogels for enhanced antioxidant properties.^29,30^ A recent study focused on improving the mechanical properties of keratin hydrogels using oxidized dopamine as a cross-linker for keratin.^4^ Another research created keratin–dopamine nanoparticles by amide bond formation as drug carriers.^31^ To the best of our knowledge, no studies on esthetic properties and skin applications have been performed on the combination of keratin and melanin.

This article describes the formation of novel keratin–melanin (KerMel) molecules that form submicron particles with tunable colors that comply with skin tones. The process is water-based, applies “green” chemistry without requiring photoinitiators or stabilizers, and has a low consumption of organic solvents. Thiol groups react with tyrosine in the presence of TYR.^20,27,32^ The KerMel reaction is based on the potential linkage between keratin’s sulfur originating in cysteine and l-dopa, inspired by pheomelanin synthesis in melanogenesis. A design of experiment (DoE) technique can identify the crucial factors for productive outcomes when pursuing novel materials or processes with numerous potential variations that can affect the output.^33^ Therefore, a DoE statistical method was used in this study to enable the KerMel reaction; the overall goal was to attain controllable pigmented keratin via starting conditions. Recently, the DoE became a useful statistical tool for research rather than the industry.^34,35^ Here, the DoE permits changing variables simultaneously and enables a lesser subset of experiments. We varied synthesis starting conditions to study their effect on molecule formation and pigmentation. First, the possibility of KerMel particle formation was analyzed, and the chemical structure was studied. Imaging helped establish a spatial arrangement model, and chromophore particles were assessed for compliance with human skin tones. KerMel may be further fabricated to hydrogels, films, coating layer, and ink for the cosmetic and biomedical industries, specifically in the makeup, skincare, and skin protection arenas.

## Experimental Section

### Materials

Raw cashmere wool was purchased from a local supplier (Ness-Ziona, Israel). l-Dopa, tyrosinase (TYR), phosphate-buffered solution (PBS), and Ellman’s reagent (5,50-dithiobis-(2-nitrobenzoic acid)) were purchased from Sigma-Aldrich (Rehovot, Israel). Dialysis membranes (MWCO 3.5 kDa) were purchased from Medicell (London, England). All solutions were formulated using deionized water (DW) or double distilled water (DDW). Human epidermal keratinocytes (PCS-200-011) and thiazolyl blue tetrazolium bromide (MTT reagent) were purchased from ATCC and Sigma-Aldrich.

### Keratin Extraction Procedure

Keratin was extracted using the Shindai method with some modifications. In brief, raw, white cashmere wool (30 g) was washed, blotted, and dried overnight in a 37 °C incubator. Then, the wool was immersed in 100% acetone for 24 h, rinsed and washed in DW, and blotted and dried overnight in a 37 °C incubator. Wool fibers were cut to about 3 mm in length and mixed with an aqueous extraction medium containing 0.5 M thiourea, 7 M urea, and 5% 2-mercaptoethanol, at 50 °C, for 72 h. The mixture was centrifuged at 11,000 rpm for 30 min, and the supernatant passed through a stainless steel sieve (#200). The resulting keratin solution was dialyzed against DW (1:50) and water changed once a day for 96 h at room temperature (RT) and then centrifuged twice at 11,000 rpm for 30 min, resulting in an approximate 20 mg/mL of keratin stock. Laemmli SDS-PAGE and the Bradford colorimetric method validated keratin purity and concentration.

### Design of Experiment (DoE)

In complex systems with several factors, it is impractical to change one factor at a time while holding all of the other factors constant. In addition, investigating the effects of one factor at a time is time-consuming and may result in a different response than an optimal response (results). The design of the experiment (DoE) method enables a systematic and efficient study of the correlation between factors and responses. With the DoE approach, all factors (variables) are simultaneously modified to determine cross-correlations between them and to maximize the statistical significance for a given number of runs. Furthermore, DoE identifies statistically significant correlations from a limited subset of tests. Correlations with confidence <95% are discarded, and the multivariate fit model is assessed for trends. Here, we study reaction starting conditions on KerMel formation. The DoE was custom-designed through JMP software version 16 with three factors chosen to identify optimal conditions.

### Design of Experiment (DoE) Factors and Responses

The design was applied for three factors: (i) reaction temperature, (ii) l-dopa:free sulfhydryl group molar ratio (also describe as l-dopa:keratin ratio), and (iii) TYR concentration. Each factor had 3 points. The temperature was 24, 32, and 40 °C, with TYR concentration in the range of 0.02, 0.035, and 0.07 mg/mL, and the l-dopa:free sulfhydryl group molar ratio, where the l-dopa ratio varied from 5, 3, 1, i.e., 5:1 is 5 times higher l-dopa to the free sulfhydryl group (in hydrolyzed keratin solution). The DoE model generated 17 formulations with different factor setups; the recipe is elaborated in Table 1. Simplified analysis presentations were demonstrated; most results are divided into three subgroups, A, B, and C, each numbered according to Table 1 nomenclature. The responses were particle size, ζ potential, two UV–vis absorbance spectra, fluorescence, and individual typology angle (ITA). Each formulation was evaluated and provided information on the dimensions, polymerized l-dopa groups, and particle aggregation of KerMel.

**Table 1:** 17 KerMel Reactions Generated by the DoE Using the Three Factors of Temperature, l-Dopa:Keratin Ratio, and TYR Concentration, and the Processed Response Input

	reaction																
	A1	A2	A3	A4	A5	A6	A7	B1	B2	B3	C1	C2	C3	C4	C5	C6	C7
Factors																	
temperature [°C]	40	40	40	40	40	40	40	32	32	32	24	24	24	24	24	24	24
TYR conc. [mg/mL]	0.02	0.035	0.07	0.07	0.02	0.02	0.07	0.02	0.035	0.07	0.02	0.07	0.07	0.02	0.02	0.035	0.07
l-dopa:keratin ratio	1	1	1	3	5	5	5	1	3	5	1	1	1	3	5	5	5
Responses																	
fluorescence emission ratio	1.40	1.30	1.14	1.58	1.19	1.66	1.22	0.91	0.86	0.90	0.87	0.84	0.85	0.88	0.92	0.90	0.91
UV–vis: ratio keratin aggregation	53	63	43	18	25	20	18	51	33	23	55	29	31	29	23	13	12
UV–vis: polymerized l-dopa	0.88	0.67	0.73	0.93	0.91	0.79	0.76	1.00	0.97	0.99	1.04	1.09	0.90	0.82	0.82	1.06	1.09
ITA [deg]	73	61	61	–51	46	21	34	38	20	22	–4	10	24	–25	–6	–20	–7
particle size [nm]	130	147	145	139	119	136	154	133	132	135	203	168	184	170	200	275	260
ζ potential [mV]	–31	–37	–30	–30	–36	–37	–43	–36	–33	–37	–34	–35	–37	–37	–40	–32	–31

### KerMel Synthesis

KerMel synthesis was performed under the DoE recipe, which determined each formulation starting condition. Fresh keratin solution was dialyzed against PBS (0.5 M, pH 6.8) for 24 h at RT. Protein concentration and free sulfhydryl were then assessed using the Bradford assay and Ellman’s reagent. The temperature (40 °C, 32 °C, or 24 °C) was set in a multiposition magnetic hot plate, and the glass flasks were located in an oil bath. All seven reactions with 40 °C were positioned in the glass flasks on the hot plate, i.e., in group A, the reaction temperature of A1 was 40 °C, the l-dopa:keratin ratio was 1:1, and the TYR concentration was 0.02 ng/mL. After the group A experiment was completed, the temperature was set to 32 °C, and all three reactions took place in group B. The group C experiment was performed after setting the temperature to 24 °C. l-Dopa was added to 10 mL of keratin solution with a known free sulfhydryl group concentration for each reaction. After 1 h of mixing, TYR stock of 2 mg/mL in DW was diluted directly into the solution. The reactions were terminated after no color changes to the naked eye or after 24 h by dialysis against DW at 4 °C for at least 3 days, with water change every 24 h followed by lyophilization, and then stored at RT. Poly-l-dopa (PLD) was prepared in a 20 mg/mL solution of l-dopa in PBS, pH 6.8, and spontaneously polymerized for 1 week.

### Characterization of KerMel

#### Fourier Transform Infrared-Attenuated Total Reflection (FTIR-ATR)

Attenuated total reflection (ATR) spectra (600–4000 cm^–1^) of lyophilized formulations were collected using an FTIR Nicolet 6700 (Thermo Scientific, Waltham, MA) equipped with an ATR accessory. l-Dopa and poly-l-dopa were measured with a KBr disc using FTIR. All data sets were normalized to 900 cm^–1^.

#### UV–Vis

Samples were diluted 4-fold in DDW, poly-l-dopa, and l-dopa, which were diluted 6-fold. Absorption spectra at 190–800 nm were measured and recorded. The data sets were normalized to 276 nm and 600 nm.

#### Dynamic Light Scattering (DLS) and ζ Potential

Samples were diluted 4-fold in DDW. Dynamic light scattering (DLS) was measured with a Malvern Zetasizer instrument. The results are reported as the mean size distribution, calculated by the Malvern software, of 10–15 measurements. The ζ potential results are reported as the mean ζ potential distribution, calculated by the Malvern software, of 100 measurements. All samples were freshly prepared from the lyophilized state and the l-dopa from its powder state, which was not fully suspended, yielding higher particle size than expected.

#### High-Resolution Scanning Electron Microscopy (SEM)

An SEM (Sirion, Thermo Scientific, Waltham, MA) was used to assess the topographical morphology of the 10 nm gold–palladium-coated samples at a 2 kV accelerating voltage. Lyophilized samples were deposited on carbon tape.

#### Cryo-Transmission Electron Microscopy (Cryo-TEM) Imaging

Three representative samples, A3, B3, and C6, and two references, keratin and PLD, were tested. Samples, excluding PLD, were prepared at the following procedure: a 3 μL drop of the sample was applied to a glow-discharged TEM grid (300-mesh Cu grid) coated with a holey carbon film (Lacey substrate, Ted Pella, Ltd.). The excess liquid was blotted, and the specimen was vitrified by rapidly plunging into liquid ethane precooled with liquid nitrogen using Vitrobot Mark IV (FEI). The vitrified samples were transferred to a cryo-holder (Gatan model 626) and examined at −177 °C using an FEI Tecnai 12 G2 Spirit TWIN TEM. The images were recorded by a 4 K × 4 K Eagle CCD camera (FEI) at 120 kV in low-dose mode. TEM imaging was applied for PLD, and a 3 μL drop was applied to a glow-discharged TEM grid (carbon-supported film on 300-mesh Cu grids, Ted Pella). After 30 s, the excess liquid was blotted, and the grids were allowed to dry for 2 h at room temperature before they were observed by an FEI Tecnai 12 G2 TWIN TEM operated at 120 kV. The images were recorded by a 4 K × 4 K FEI Eagle CCD camera.

#### Separation of Nanoparticles by Gel Electrophoresis

Electrophoresis was performed in a 1% agarose gel. Each sample was supplemented with 10% glycerol, while poly-l-dopa was diluted 6-fold. The gel was run at 100 V, in tris-borate-EDTA (TBE), pH 10, for 1 h, and then exposed to UV light at 365 nm. Gels were stained with Coomassie blue for 1 h and then incubated in DW at RT for 24 h.

#### Fluorimetry

Samples were diluted 4-fold in DDW, and fluorescence was measured using a fluorometer (Cary Eclipse, with polarized light accessory) at an excitation wavelength of 320 nm. Emission was measured at 400–600 nm. Poly-l-dopa and l-dopa were diluted 6-fold.

#### Color Analysis

The KerMel chromophores retain color after synthesis termination and cleaning. The colors were recorded and measured using digital images in two states, first, the KerMel solution, and second, as films. The film was fabricated by casting 10 mg/mL of dissolved lyophilized KerMel in DW solutions. The colors of the films were digitized by translating them to RGB values using the eyedropper toolbar in PowerPoint Microsoft; then, computational calculations of color values were predicted for the case of mixing two colors. All 17 obtained values were mixed in pairs, and their color scheme was assessed in eight points. The computational calculation was done by the Java script algorithm in the React framework, in n choose k- no repeats and no order, resulting in 1053 different colors.

#### KerMel Cytotoxicity

Human epidermal keratinocytes at passage six were used to evaluate the biocompatibility and cytotoxicity of the fabricated nanoparticles. The keratinocytes were cultured in dermal cell basal medium, supplemented with specific growth factors required for keratinocytes, including apo-transferrin (5 mg/mL), hydrocortisone hemisuccinate (100 ng/mL), l-glutamine (6 mM), epinephrine (1.0 mM), rhTGF-a (0.5 ng/mL), bovine pituitary extract (0.4%), and rh-insulin (5 mg/mL). Antibiotics (streptomycin (10 μg/mL) and penicillin (10 Units/mL)) were also added to minimize any risk of contamination. The cells were maintained in a sterile humidified incubator at 37 °C and 5% CO_2_. Human keratinocytes were seeded in 96-well tissue culture plates at 4000 cells/well density. After 24 h of culture, filter-sterilized samples A3, B3, C5 and keratin were added in a range of concentrations (5, 2.5, 1, 0.5, 0.25, 0.1, and 0.05 mg/mL). The samples were incubated with the cells for 24 h before cytotoxicity was evaluated using the 3-(4,5-dimethylthiazol-2-yl)-2,5-diphenyltetrazolium bromide (MTT) assay, following the manufacturer’s protocol.

#### KerMel Statistics

Data were analyzed with GraphPad Prism 9.3.1 software. The results of the DoE were plotted as repeats due to trend detection and ease of results display using one-way analysis of variance (ANOVA). Statistical significance was determined with a *p*-value ≤ 0.05.

## Results and Discussion

**Figure 1:**
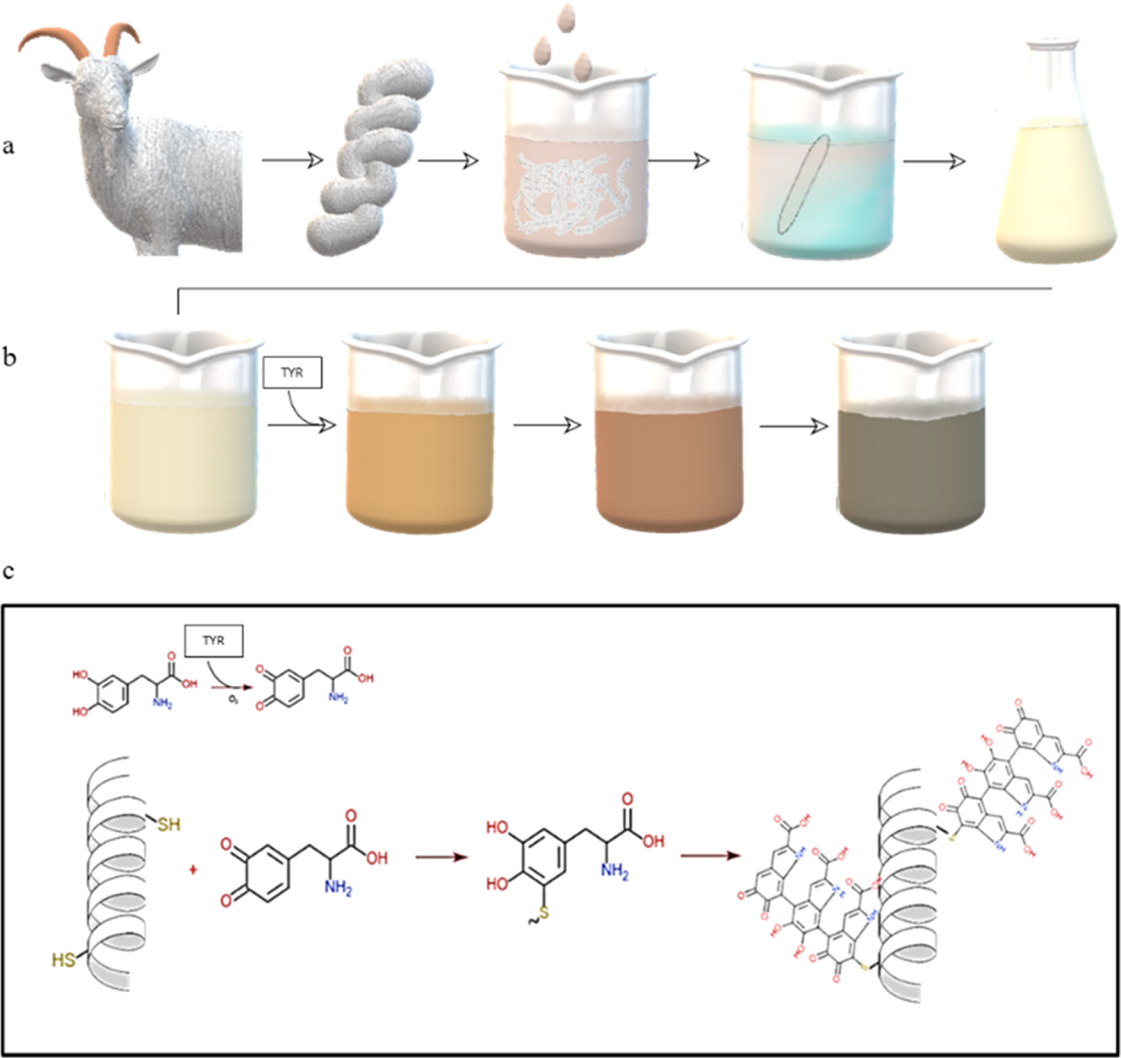
KerMel synthesis process: (a) keratin extraction process describing the hydrolysis of cashmere wool by aqueous extraction solution, following dialysis that yields keratin solution followed by (b) addition of l-dopa and TYR (c) Suggested KerMel mechanism, depicting the polymerization of melanin as a function of l-dopa. polymerization to melanin l-dopa is oxidized to dopaquinone, continuing to cysteinyldopa, when thiol is introduced. Linked cysteinyldopa spontaneously reacts with free l-dopa, resulting in polymerized melanin residues on the keratin backbone.

KerMel was formed via a reaction between keratin and l-dopa in the presence of TYR. More specifically, keratin from cashmere wool was extracted using the Shindai method^36^ and reacted immediately with l-dopa, which resulted in notable pigment development, which evolved from light pink to bright orange and ended with brown to gray shades (Figure 1a). The nature of keratin under hydrolyzed conditions leads to a high probability of possible disulfide, hydrogen, hydrophobic, and ionic bond forming.^11^ Keratin’s functional groups, including the thiol groups, are expected to be grafted by l-dopa, which further polymerizes to poly-l-dopa (PLD) or melanin. Cysteine, which comprises approximately 8% of the keratin backbone, carries a highly nucleophilic side chain, sulfhydryl. Therefore, this is a preferable candidate to react with l-dopa, likewise the pheomelanin pathway.^13,14,37^Figure 1b presents the suggested mechanism, in which l-dopa is oxidized to dopaquinone and then continues to cysteinyldopa when a thiol is introduced. The linked cysteinyldopa then reacts with free l-dopa, resulting in polymerized melanin residues on the keratin backbone (Figure 1c). The suggested mechanism relies on the Michael addition, Schiff base, or aryl–aryl coupling.^4,26,38^ In the pheomelanin pathway, benzothiazine and benzothiazole intermediates are formed and are not expected to appear in the KerMel synthesis. The linked cysteinyldopa constraints the dopaquinone to further polymerize to the intermediates and is expected to result in the heterogeneous aggregation of intermediate oligomers.^20,27^ In pheomelanin, the cysteine presence dictates pheomelanin formation. Therefore, this reaction’s cysteine concentration on the keratin backbone is a limiting factor and depends on the keratin type and the extraction method; high cysteine content and reactive cysteine functional group are expected to achieve different results. The concentration of free sulfhydryl in the KerMel reaction is over two magnitudes higher than required for the oxidation of cysteinyldopa toward pheomelanin.^18^ While side reactions, such as tyrosine polymerization originating in the keratin backbone,^4,26^ can occur, their rate is estimated to be four times slower due to the lower tyrosine concentration compared to cysteine. In melanogenesis, the oxidation of tyrosine to dopachrome results in a red hue.^39^ The KerMel reactions show a similar color development, indicating that the KerMel reaction occurred and that the TYR activated the l-dopa.

**Figure 2:**
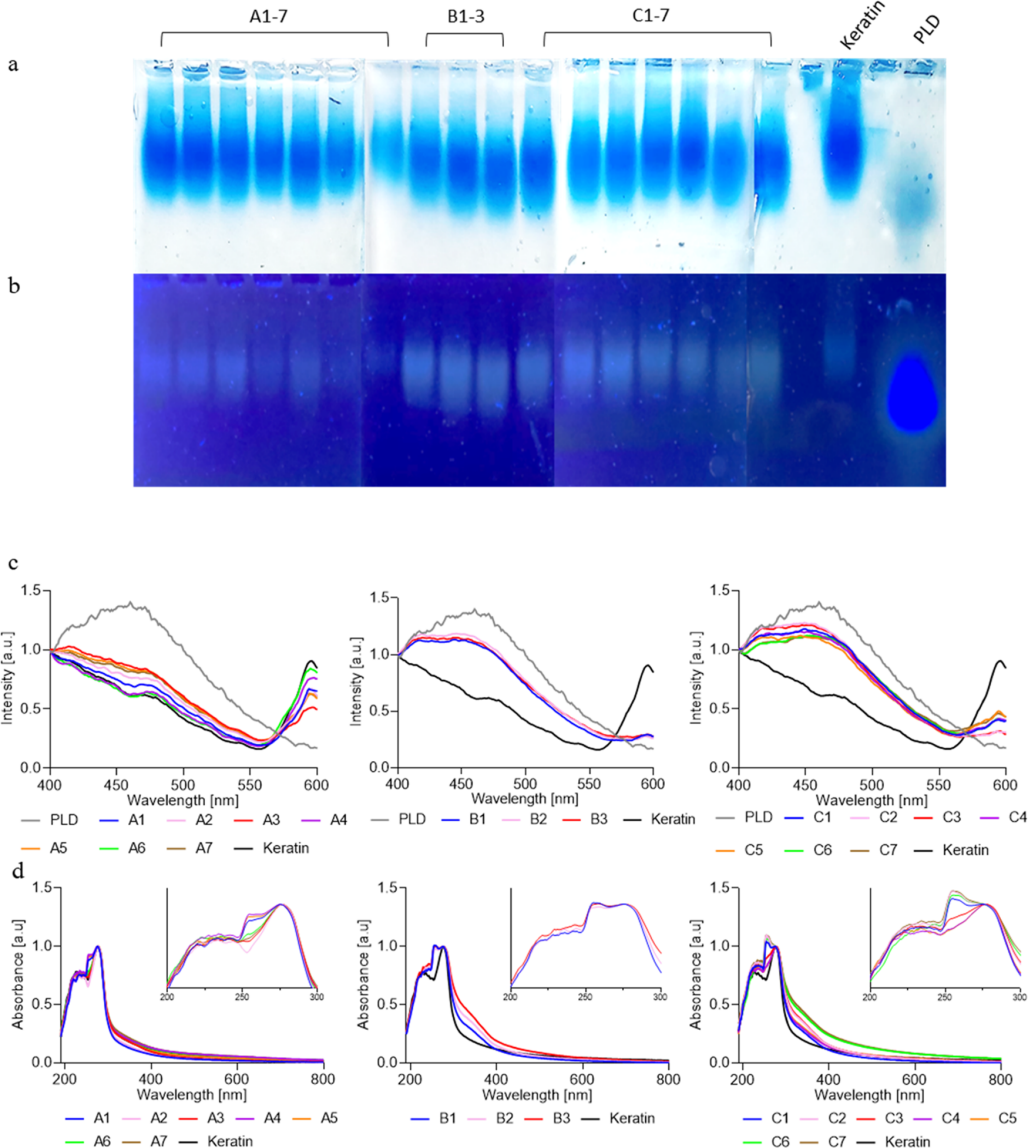
Agarose gel electrophoresis was loaded with samples and analyzed for protein and PLD presence. (a) Agarose gel stained with Coomassie brilliant blue for protein detection. (b) Agarose gel under UV light to ensure no phase segregation in KerMel. (c) Fluorescence spectrum of KerMel vs keratin (black line) and PLD (gray line). Reactions B and C resemble the poly-l-dopa wave structure. (d) UV–vis spectra, a keratin peak at 276 nm, and a new peak at 254 nm indicated new aromatic bonds. The magnification shows a profile between 200 and 300 nm.

To gain a comprehensive understanding of the reaction and critical parameters, we followed the design of experiment (DoE) tool. DoE is a statistical method that aids in narrowing the variation when approaching new and complex systems. It analyzes the dependence of a given variable in a system and its significance. Eumelanin and pheomelanin ratios depend on the availability of TYR, tyrosine, and cysteine.^18^ Here, the KerMel reaction replaced tyrosine with l-dopa and cysteine with the keratin backbone. KerMel reaction is highly affected by the starting conditions. Based on preliminary experiments and a literature review, the DoE starting condition parameters were selected. The temperature was in the range of 24–40 °C, TYR concentration was in the range of 0.07–0.02 mg/mL, and the l-dopa: keratin ratio was 1:1, 3:1, and 5:1. Keratin concentration and pH levels were fixed to 20 mg/mL and 6.8, respectively. The DOE platform generated 17 reactions described in the Experimental Section. In order to analyze and present the results, the reactions were classified into three groups, A, B, and C, and each group is numbered A1-A7, B1-B3, and C1-C7, respectively (see Table 1). The typical l-dopa polymerization occurred during the reaction, exhibiting the development of colors associated with this process (transparent-red-brown), as described in the literature.^20,32,40^ The study examined possible reactions between keratin backbone and l-dopa for the development of melanin, with conditions similar to those in the human body. Melanin analysis is challenging due to a complex reaction to begin with.^22^ Keratin has multiple functional groups and thus multiple reactive sites, which combined with limited prior knowledge of the keratin–dopa reaction is even harder to analyze. Therefore, several analytical methods were combined to shed light on the KerMel reaction. First, the assessment of whether the KerMel reaction is compatible with keratin and does not cause any phase separation or parallel reactions is presented in Figure 2. The obtained KerMel particles were evaluated in an agarose gel based on a method for nanoparticle separation by gel electrophoresis based on their size and shape.^41^Figure 2a shows a Coomassie brilliant blue-stained gel, demonstrating the presence of keratin in all final KerMel particles. After exposing the same gel to UV light in Figure 2b, the reactions and the references showed luminescence. Interestingly, the KerMel reaction luminescence bands appeared between the reference bands, pointing to the KerMel formation; in case of no reaction, two distinct bands would have appeared. L-Dopa alone was not detected, while the polymerized l-dopa was detected under UV light, as described elsewhere. PLD is a smaller molecule than hydrolyzed keratin; therefore, it is transported further in the gel. As the KerMel particles exhibited luminescence under UV light, the reaction intensities were measured with a fluorometer (Figure 2c). PLD showed a fluorescence absorbance peak at 460 nm, while keratin showed absorbance across the entire measured spectrum (400–600 nm). Spectra were normalized to 400 nm, focusing on the resemblance to PLD to assess the presence of polymerized groups of l-dopa. As the fluorescence spectra were similar to that of PLD, we can claim that typical structures, such as indole, were developed in the KerMel formation. The results are displayed and classified into three fluorescence spectra A, B, C. Group A profile is similar to keratin. Therefore, it can be concluded that fewer aromatic rings arose in reactions in group A. In contrast to group A, groups B and C followed the PLD pattern, implying similarity between their structures. The fluorescence profile of groups B and C is not identical to the PLD, and the reason might be because of another side chain from other interactions, such as with tyrosine, that was proved to oxidize in the presence of TYR in a tripeptide.^27^ The PLD emission shifted toward 450 nm rather than that reported on l-dopa polymerization to PLD at 460 nm peak.^42−44^ The different polymerization conditions and the other functional groups within the keratin backbone, such as thiol, could have caused the shifting. A PLD signal was established in most of the KerMel reactions; we sought to comprehend the existence of new connections that will vouch on a novel molecule rather than blend or coat on the keratin backbone. Figure 2d shows the UV–vis spectra of groups A, B, and C. UV–vis absorbance analysis identified distinct peaks for keratin amino acids and PLD molecules at 276 and 280 nm, respectively. Most of the KerMel particles showed a new peak at 254 nm, reflecting a chromophore that, when attached to functional groups such as −OH, −NH_2_, and −SH, shifted to a longer-wavelength auxochrome. Such behavior can imply characteristics of aromatic conjugation molecules bearing elements such as sulfur, lacking in keratin and PLD. In group A, A2 and A3 lacked this peak, which appeared in most reactions in groups B and C spectra. However, it was significantly lower in C3, C4, and C5. In addition, when observing in a 200–300 wavelength magnification of A, B, and C, the ratio between the absorption of 254 nm and 276 nm peaks changes between the groups. Group A had a higher peak at 276 nm, group B had equal peaks between 276 nm and 254 nm, and group C had a higher peak at 254 nm. A higher peak at 254 nm points to ample conjugated aromatic rings, suggesting the grafting of l-dopa. The assumption that l-dopa occupied cysteine groups was examined by secondary analysis of UV–vis (normalized to 600 nm). The UV–vis spectra showed an interesting absorbance trend at 276 nm (Figure S1). Keratin aggregation is evaluated by hyperchromicity at 276 nm, suggesting that free thiols can oxidize to cysteine form.^45^ The sample with a lower l-dopa:keratin ratio exhibited more keratin aggregation, regardless of the reaction temperature. As the l-dopa:keratin ratio increases, there is a possibility that the thiol–catechol reaction is preferred due to the spontaneous nucleophilic attack of quinones by the thiol group.^46^ However, increasing that ratio resulted in more side reactions, such as amino groups or PLD.^26,38^

**Figure 3:**
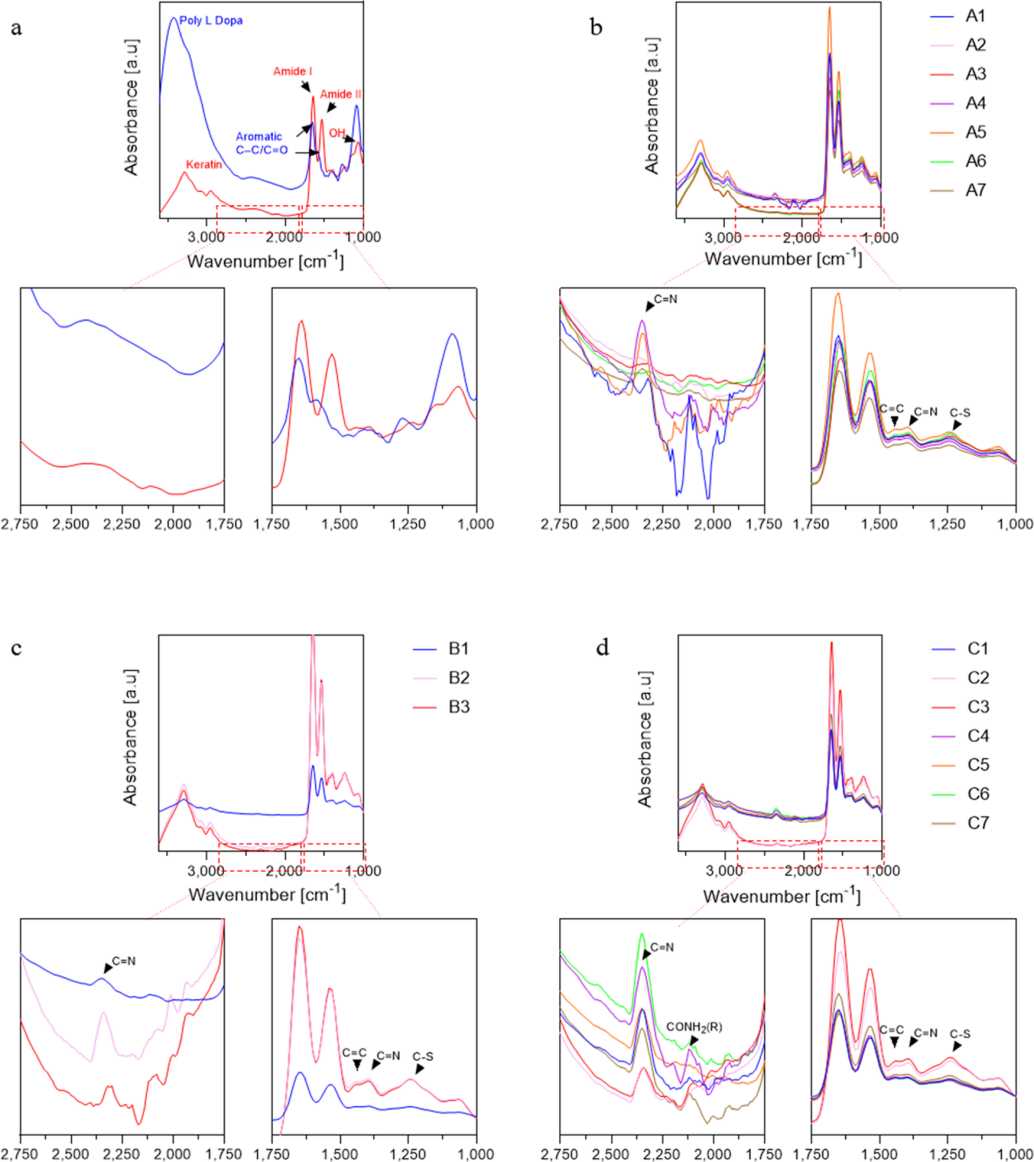
Fourier transform infrared-attenuated total reflectance (FTIR-ATR) with two magnifications of 1000–1750 and 1750–2750 cm^–1^. (a) Keratin and poly-l-dopa, magnifications of the peaks at 1750–3000 and 1000–1750 cm^–1^. (b) KerMel A. The peak at 1245 cm^–1^ is assigned to C–S and 1395 and 1450 cm^–1^ to aromatic C=N and C=C, respectively. The peak at 2345 cm^–1^ is a new peak of aromatic C=N. (c) KerMel B. (d) KerMel C. The peak at 2100 cm^–1^ reflects a CONH_2_(R) belonging to l-dopa that was not fully polymerized.

The ATR analysis provided a better understanding of the nature of the functional group. The primary amide of keratin was identified at 1640 cm^–1^, secondary amides at 1530 cm^–1^, and a primary amine at 1070 cm^–1^ (Figure 3a). The peak at 3290 cm^–1^ is ascribed to −NH and −OH. PLD showed similar spectra and aligned with a previous report.^28^ The peaks at 1650 and 1585 cm^–1^ could be attributed to the C–C and C=O in PLD aromatic rings. The peak at 3420 cm^–1^ is assigned to −NH and −OH. Figure 3b–d represents groups A, B, and C. Group A peaks at 1245 cm^–1^ were ascribed to C–S^47^ and 1395 and 1450 cm^–1^ to aromatics C=N and C=C, respectively. The peak at 2345 cm^–1^ implies a new C=N bond that can indicate a new aromatic ring formed during the l-dopa grafting and polymerization on the keratin backbone. The magnification of 1000–1750 cm^–1^ revealed an increase at the peak of C–S compared to the primary amine of keratin, which suggests the disappearance of the latter group for the creation of indole groups (peaks 1395 and 1450 cm^–1^). The increase in C–S suggests that new bonds were created. This trend was also observed with groups B and C. Reactions A1, A4, A5, B3, C3, C4, C6, and C7 showed a prominent additional peak at 2100 cm^–1^ that can be ascribed to CONH_2_(R), indicating that l-dopa did not fully polymerize. However, it also showed the C=N peak. The new C–S peak was not noted in reactions A1 and A6, which showed only slight polymerization. For all samples, the peak intensity at 3290 cm^–1^ increased dramatically, likely due to an addition of a hydroxyl group in the newly synthesized catechol.^31^

**Figure 4:**
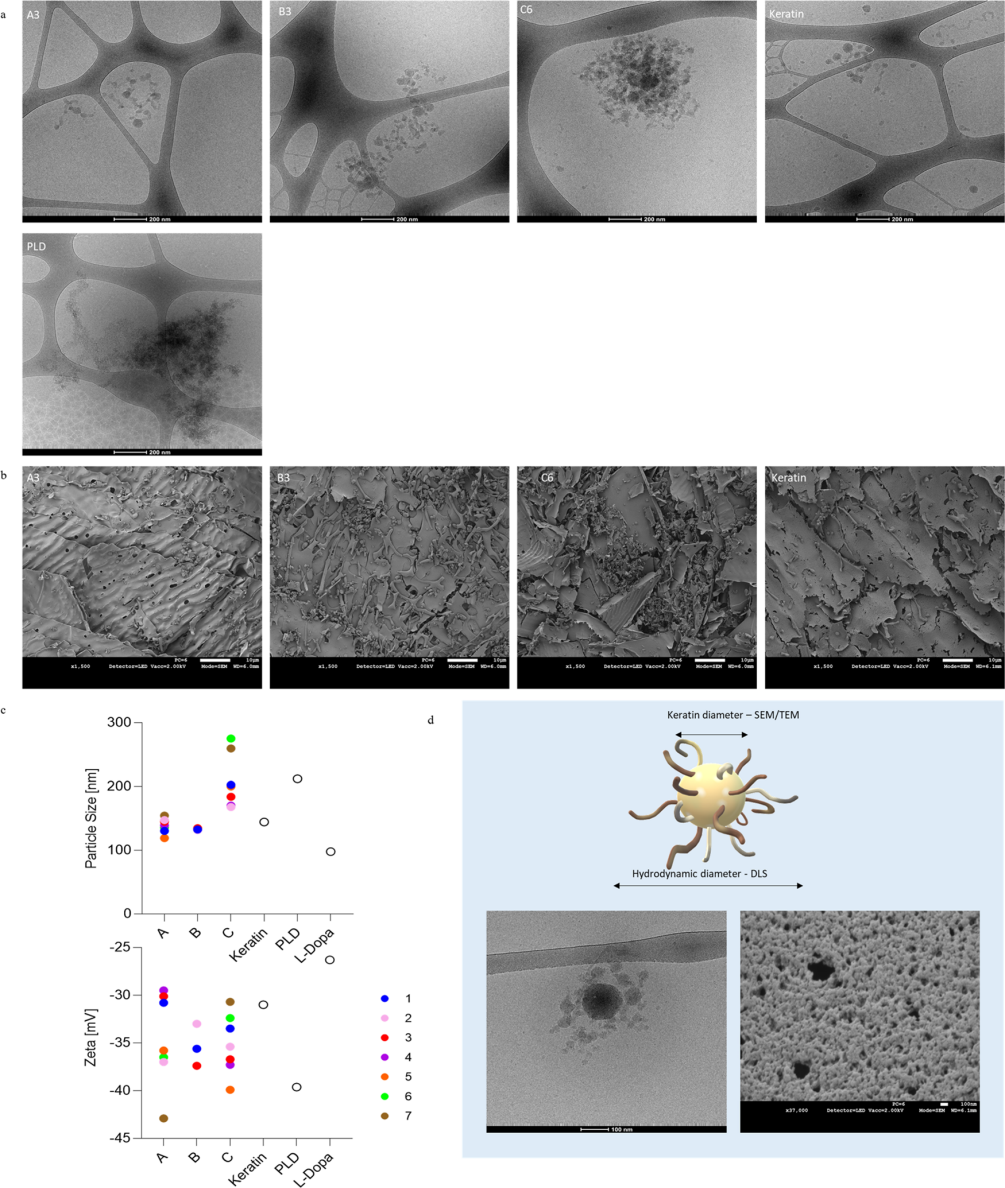
Imaging of KerMel reactions. (a) Cryo-transmission electron microscopy (cryo-TEM) imaging of a representative KerMel sample, A3, B3, and C6. (b) SEM images of lyophilized KerMel show a typical layered structure lacking a grainy assembly in keratin-only samples. (c) The particle size of groups A, B, and C ranges between 100 and 300 nm (up), and the ζ potential of groups A, B, and C ranges between −30 and −45 mV, demonstrating their stability in suspension (down). (d) Suggested model of KerMel based on SEM/TEM and DLS results.

Cryo-TEM, SEM, and DLS analyses were performed to characterize the KerMel’s particle morphological structure. The cryo-TEM in Figure 4. showed that most keratin particles were ≤100 nm (diameter) round structures, with some small interconnected strings between two or more particles. PLD was reported to be 50 nm under spontaneous oxidization;^42^ in the present work, PLD clusters were aggregated in the range of 100–200 nm, as previously reported.^28,43,44^ A reaction sample from each group A, B, and C (A3, B3, and C6) was examined and contained a similar structure of round keratin-like particles surrounded by strings that appeared to be interconnected. There was an apparent increase in the cluster size, structure density and compactness, and aggregation from A to C, while A showed the lowest numbers of round shapes and strings forming the structures and C showed the highest. The SEM images of lyophilized KerMel (Figure 4b) showed the layered keratin structure, as in many cases in nature and as previously described.^4,48−50^ For keratin, the surface was rough and contained round nanoparticles with a diameter of ≤100 nm. The A3 structure showed a similar layered form, lacking the porous morphology, while the surface appeared continuous and smooth. The same surface morphology was noted in all sample magnifications (Figure S2). However, the structure was compromised, from layered and petals at A to random and notch at C. The change in the disrupted crystallinity could explain the structure and enable other arrangements of the keratin backbone due to hydrogen bond elimination by tyrosine oxidation^51^ or the melanin grafting that further destroyed the organized and packed layered structure of keratin, as shown in similar work.^4^

The size and ζ potential of the fabricated KerMel were measured to determine their physical and electric potential (Figure 4c). The particle size varied from 100 to 300 nm. There was a noticeable trend between groups A, B, and C; A had the smallest particle size ranging between 114 and 155 nm, B in the 131–135 nm range, and C had the highest range of 168–260 nm and consisted of cryo-TEM results. All particles, excluding C6 and C7, were higher than l-dopa. In addition, group C was larger than keratin, suggesting the creation of KerMel. ζ Potential for all groups A, B, and C was within the range of keratin and PLD (−30 to −40 mV, respectively), which indicates that KerMel particles are stable in a solution. In addition, it is supporting evidence for grafted l-dopa, where l-dopa holds a ζ potential of −26 mV. Following the observed characteristics and a primary round particle surrounding structures organized as chains or loops, we suggest a KerMel spatial arrangement model: a primary keratin particle with grafted l-dopa and smaller keratin particles (Figure 4d). In Figure 4d, bottom left, a magnification of the cryo-TEM image of a single structure that supports the model was spotted. Keratin particles tend to aggregate, and one study suggested a similar all-keratin model of central particle and chain and loop arrangement.^45^ Here, we support this model and add the grafted l-dopa that serves as a bridge between several keratin particles. We can assume that a hydrodynamic diameter depends on the keratin and grafted l-dopa chains.

**Figure 5:**
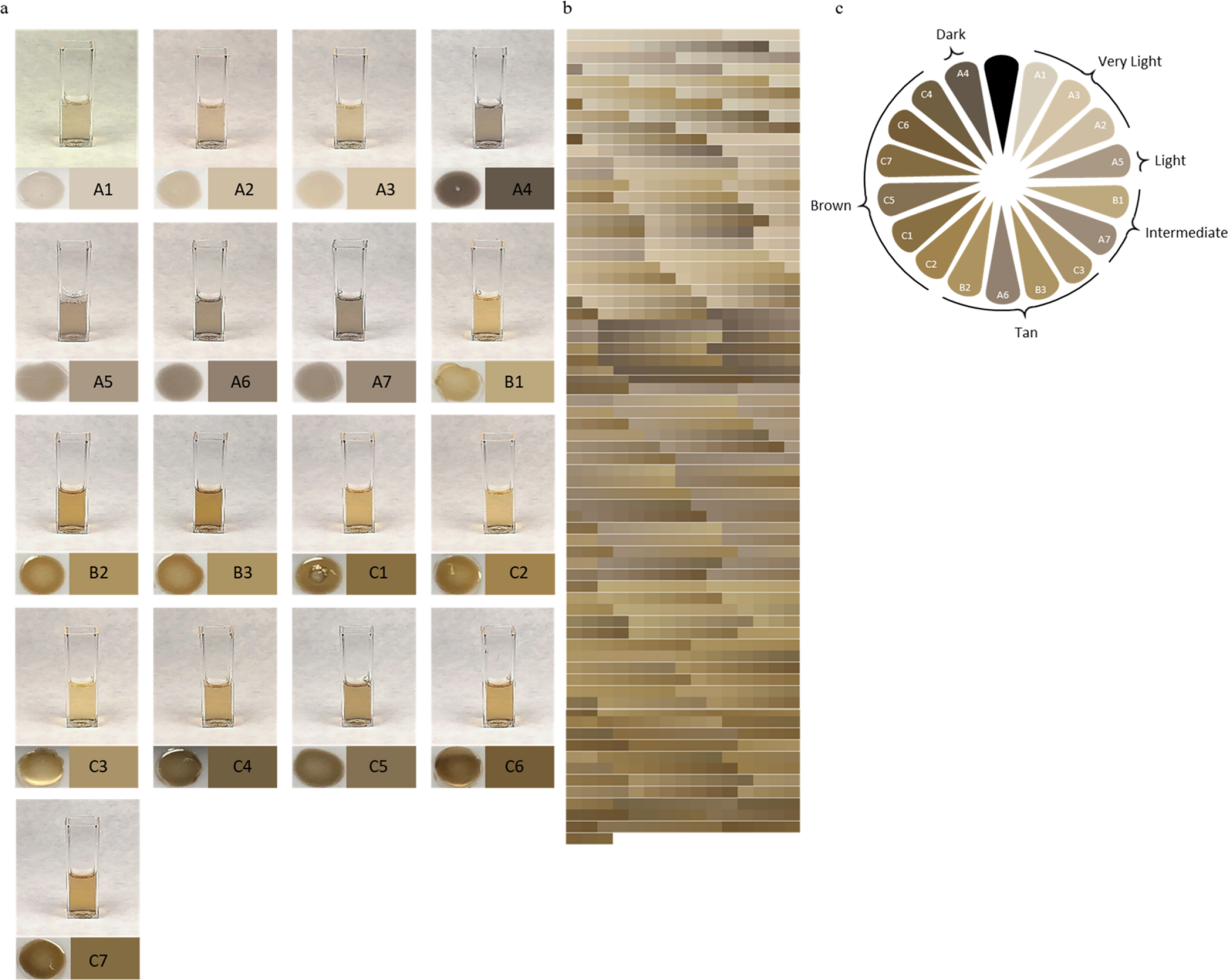
KerMel reaction colors. (a) KerMel reaction digital images of solution (top), digital images of KerMel film (left bottom), and digitized color (right bottom). (b) Computational calculation of 1053 different KerMel colors. (c) Individual typology angle (ITÅ), representing the human skin color range of KerMel digitized colors.

The KerMel particles, are also referred as pigmented keratin because their colors were attained by modifying the reaction conditions, with colors falling in the tan and brown ranges (Figure 5a). Group A leaned toward gray, resembling eumelanin. Groups B and C generally had a yellow-orange appearance resembling pheomelanin. In each group, the color was darker, with higher l-dopa:keratin ratios. The color development within each group reflects the polymerization of l-dopa after reacting with keratin functional groups. In order to explore the color spectrum that can be achieved from the obtained KerMel particles, computational calculations of color values were predicted, resulting in 1053 different colors (Figure 5b). When analyzing KerMel’s resemblance to authentic skin tones, all six skin tones were present, i.e., the obtained KerMel colors cover the entire skin tone range (Figure 5c). Group A, with a low l-dopa:keratin ratio, dominated the very light group, whereas group C dominated the brown group.

**Figure 6:**
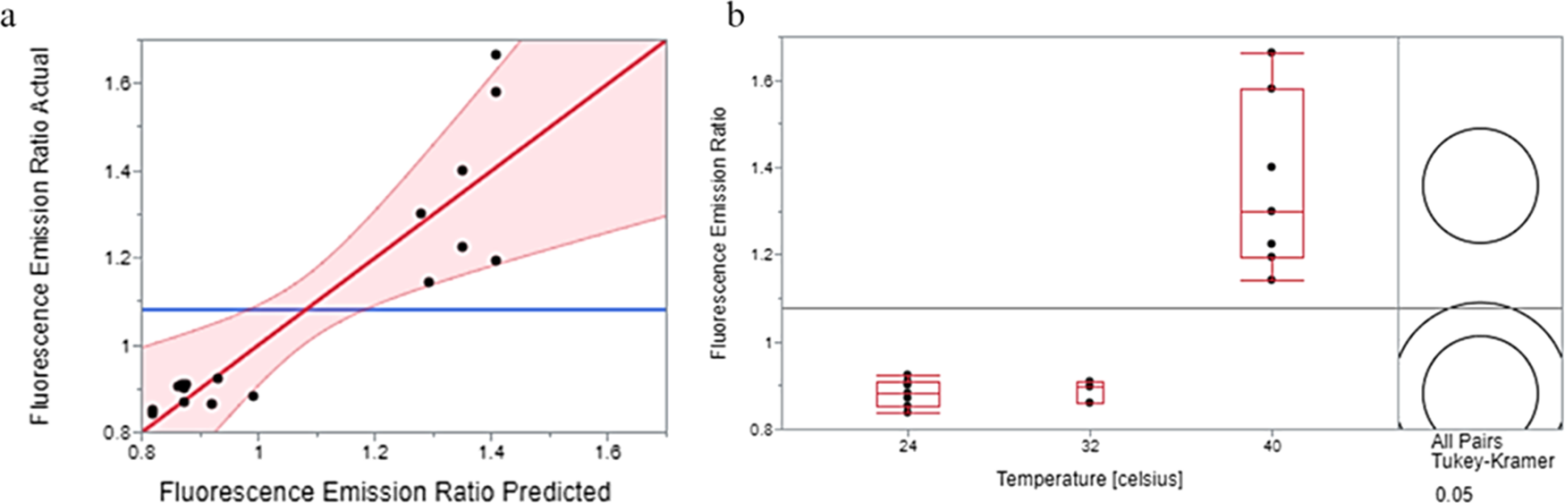
Data of the fluorescence emission responses of DoE. (a) Actual by-prediction plot generated by the DoE of fluorescent emission, demonstrating the model’s credibility of 83% (*p* = 0.0025). (b) The Tukey–Kramer test of fluorescent emission by temperature shows results in significant effects between 40 and 32 °C (*p* < 0.0001) and between 40 and 24 °C (*p* = 0.0004).

The DoE permits changing all variables simultaneously and enables a lesser subset of experiments. We varied synthesis starting conditions to study their effect on molecule formation and pigmentation. Reaction parameters of temperature, l-dopa:keratin ratio, and TYR concentration were selected as the variables, while pH and keratin concentration were fixed. The model was calculated based on the experimental observation and aimed to determine KerMel particle formation. Table 1 exhibits the mentioned reaction parameters and the results (responses). The results were processed to the six responses: (1) the fluorescent emission of the KerMel wavelength ratio between 400 and 460 nm, keratin and PLD maximal peaks, respectively, estimating the resemblance to keratin or PLD. (2) The UV–vis in wavelength at 254 nm formation and its intensity indicates new auxochrome on polymerized l-dopa. (3) The UV–vis wavelength at 276 nm formation and its intensity indicates keratin aggregation. (4) The calculated ITÅ values of KerMel and the higher ITÅ value reflect brighter skin tone. The fifth and sixth responses were particle size and ζ potential. Each response generated data sets, including the actual by-prediction plot, which provides a visual evaluation of model fit that reflects the variation due to random effects. Figure 6a presents the actual prediction plot of fluorescent emission, demonstrating the model’s credibility of 83% (*p* = 0.0025). From the actual prediction plot, we can conclude the reliability of the responses. The UV–vis indicating keratin aggregation showed the highest model credibility (90%, *p* = 0.0002). Following fluorescence emission and particle size (83%, *p* = 0.0022), the ITÅ (75%, *p* = 0.0121) and UV–vis indicated polymerized l-dopa (38%) and ζ potential (18%) with no significance. Figure 6b depicts the Tukey–Kramer test of fluorescent emission by temperature, which shows that the temperature has significant effects between 40 and 32 °C and between 40 and 24 °C. Lower temperature reactions showed more resemblance toward PLD and contained more polymerized l-dopa. The UV–vis indicating keratin aggregation had l-dopa:keratin ratio as a leading factor and exhibited a significant effect between the 1:5 ratio (*p* = 0.0002) and the 1:3 ratio (*p* = 0.0206). A lower l-dopa:keratin ratio promotes particle formation. At a 1:1 l-dopa:keratin ratio, l-dopa might prefer to be grafted on keratin rather than to PDA; this explains the increased particle formation in samples with lower l-dopa:keratin ratios. The particle size significant effect was the temperature between 24–32 °C (*p* = 0.0053) and 24–40 °C *p* = (0.0013); as it increased, the particle size increased. The ITÅ main factor was the l-dopa:keratin ratio between 1 and 3 (*p* = 0.0309). Higher l-dopa:keratin ratio promoted darker colors of the KerMel reactions. It can be explained by the free L-Dopa excess that can be polymerized on the grafted keratin backbone, creating longer melanin-like chains. The UV–vis indicating polymerized l-dopa showed that the temperature was the main factor and exhibited a significant effect between the 24 and 40 °C ratios (*p* = 0.0225). A lower reaction temperature promotes new aromatic groups. Finally, ζ potential had an insignificant impact on KerMel formation, likely due to the presence of PLD and keratin, both of which hold a low and stable ζ potential. All factors’ significations are summarized in Table 2. All responses to actual versus prediction plots and Tukey–Kramer tests are shown in Figures S3 and S4, respectively. This DoE model indicates that (i) lower temperature promoted KerMel formation, (ii) there was a weaker dependence of the l-dopa:keratin ratio, where a higher ratio promoted KerMel formation, and (iii) the TYR concentration was redundant, unlike in the melanogenesis pathway in the human body; the KerMel reaction executed in a controlled environment, meaning that the TYR concentration does not contribute or detract the KerMel formation. TYR oxidation is solely responsible for the oxidation of l-dopa to dopaquinone. Therefore, it did not influence the reaction with the thiol, which is a nonenzymatic reaction.^39^ The formation of new aromatic groups, i.e., increased polymerization of l-dopa, was primarily impacted by temperature, which, when increased, was associated with less polymerization. The formation of new particles was influenced by the l-dopa:keratin ratio, with lower ratios promoting physical or chemical bonds. The optimized reaction for grafted l-dopa on the keratin backbone should be synthesized under lower temperatures and a mid-l-dopa:keratin ratio.

**Table 2:** Response Significance as a Function of the Factors by the Tukey–Kramer Test^a^

responses	temperature [°C]	l-dopa/keratin ratio	TYR con. [mg/mL]
fluorescence emission ratio	+	–	–
UV–vis ratio keratin aggregation	–	+	–
UV–vis ratio polymerized l-dopa	+	–	–
ITA [seg]	–	+	–
particle size [nm]	+	–	–
ζ potential [mV]	–	–	–

a (+) indicates on significance; (−) indicates on no significance.

**Figure 7:**
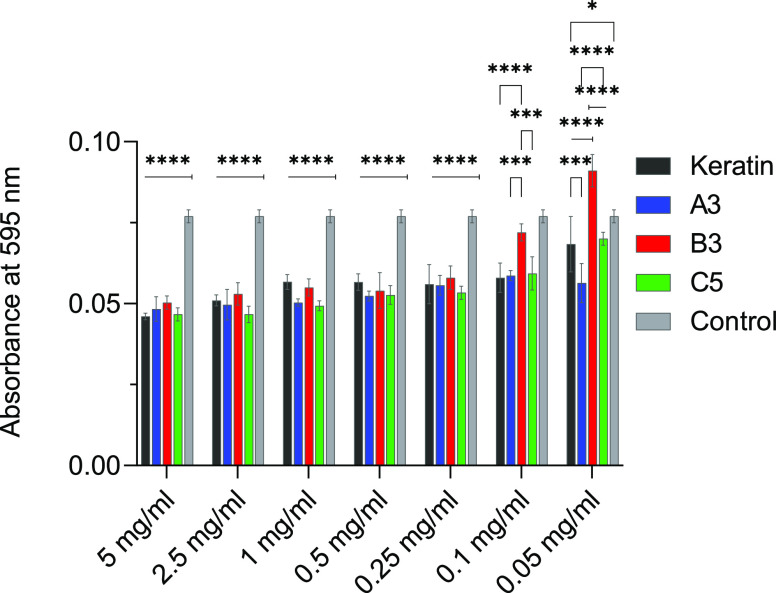
Evaluation of the cytocompatibility and cytotoxicity of KerMels using the MTT assay. KerMel B3 was more cytocompatible than control (tissue culture plastic) at concentrations of <0.05 mg/mL.

As cosmetics or medical devices were the target applications for the fabricated KerMel, cytocompatibility and cytotoxicity were investigated. The reaction samples covering a range of concentrations (5–0.05 mg/mL) were randomly selected (A3, B3, C5) and incubated with the human epidermal keratinocytes. Cell viability was assessed using a metabolic activity-based MTT assay (Figure 7). At higher concentrations (5 mg/mL), all fabricated KerMel samples and keratin induced nearly 40% cytotoxicity compared to the control (tissue culture plastic). However, at 0.1 mg/mL, the cytotoxicity of A3, C5, and keratin samples dropped to nearly 25%. B3 appears to be only 7% cytotoxic at 0.1 mg/mL and significantly increases cellular proliferation at 0.05 mg/mL compared to the control. Herein, the B3 sample is nontoxic and cytocompatible at a lower concentration (<0.05 mg/mL). Moreover, B3 is two times more cytocompatible than the synthesized melanin particles.^19^ The keratin backbone contains RGD and LDV amino acid sequences important to cell adhesion^30^ and can promote KerMel cytocompatibility.

## Conclusions

For the creation of pigmented keratin, a novel keratin–melanin molecule was developed, keratin, the material that preserves cell integrity in human skin and that is under study and development for regenerative medicine and cosmetic applications was reacted with melanin building blocks. The KerMel particle presents tunable pigmentation replicating human skin tones. The melanin-grafted keratin particle was generated by polymerizing l-dopa on the keratin backbone using a water-based enzymatic reaction inspired by the melanogenesis pathway. Furthermore, the study focused on the chromophores that can be achieved when changing the reaction starting conditions. A design of experiment (DoE) statistical method was used in this study to guide decision-making regarding the conditions for the KerMel reaction. Here, the DoE permitted the simultaneous modification of 17 reactions. The starting conditions of temperature, TYR concentration, and l-dopa: keratin ratio were varied and studied for their effect on particle formation and pigmentation. KerMel reactions yielded submicronic particles in the 100–300 nm range, featuring a new chemical bond and pigmentation range. The particles exhibited a distinguishing spatial arrangement of central keratin particles surrounded by grafted chains and loop structures. The reaction between dopaquinone and a thiol group that originated in keratin likely drove the grafting of melanin on the keratin backbone. The suggested mechanism oxidized l-dopa to dopaquinone and continued to cysteinyldopa upon introducing a thiol. The intermediate then spontaneously reacted and polymerized with free l-dopa residues, resulting in melanin grafted on the keratin backbone, generating heterogeneous aggregation of particles. Chemical analysis using FTIR-ATR identified a peak at 2345 cm^–1^, which implied a new C=N bond in the aromatic ring that formed during the polymerization of the l-dopa to melanin. Augmentation of an additional peak at 1250 cm^–1^ can be assigned to C–S, suggesting a new bond between dopaquinone and a thiol. Fluorescence microscopy indicated PLD on the keratin backbone, and the UV–vis identified modified auxochrome that can occur due to l-dopa grafting. KerMel specimens were cast into a thin film for color analysis. Colors within the ITÅ range were observed. Higher l-dopa content and higher temperature conferred darker colors, attributable to PLD polymerization. At 0.1 mg/mL, the cytotoxicity of KerMel dropped to as low as 25%, which can be considered cytocompatible. Through the DoE, it was discovered that temperature is the primary variable that affects KerMel formation; higher temperatures favored PLD formation, while lower temperatures favored l-dopa grafting. In other words, higher temperatures promoted eumelanin-like synthesis, whereas lower temperatures promoted pheomelanin-like creation. KerMel formation was also dependent on the l-dopa:keratin ratio. We also found that unlike the melanogenesis pathway in the human body, in the KerMel reaction, TYR concentrations seemed insignificant, with no impact on most tested parameters. Here, we showed that we could obtain pigmented keratin by modifying the starting condition. Keratin is currently used in cosmetics, such as skincare and hair products. Therefore, KerMel particles can be incorporated into cosmetics and regenerative skin as a functionalized pigment. The KerMel color range can be remarkably improved once it covers more pigments, such as pure red and orange. In addition, fabricating films or coatings from KerMel can highly affect the obtained color and will depend on several parameters such as thickness and could be considered as a structural color. Future investigation should focus on KerMel-based formulation for ink or coating, mainly in the cosmetic industry, as a sustainable pigment.

## Supporting Information Available

UV–vis spectra of groups A–C, a keratin peak at 276 nm, SEM magnification image of KerMel, actual by-prediction plot, and Tukey–Kramer tests (PDF)

## Abbreviations

KerMel: keratin–melanin
TYR: tyrosinase
PLD: poly-l-dopa
