# Supplementary material for: In Situ Synthesis of Keratin and Melanin Chromophoric Submicron Particles
Source: ACS Omega. 2023 Jul 5;8(30):26762–74. doi: 10.1021/acsomega.3c00189 (PMC10398706; doi:10.1021/acsomega.3c00189)
Supplement: Supplementary file 1 — ao3c00189_si_001.pdf [file ao3c00189_si_001.pdf]

In-situ Synthesis of Keratin and Melanin Chromophoric Submicron Particles

Supporting Information

Chen Nowogrodski *†, *Yaniv Damatov* †, *Sunaina Sapru* † *and Oded Shoseyov* †

†Plant Molecular Biology and Nano Biotechnology, Faculty of Agriculture, Food and Environment, The Hebrew University of Jerusalem, Rehovot 7610001, Israel

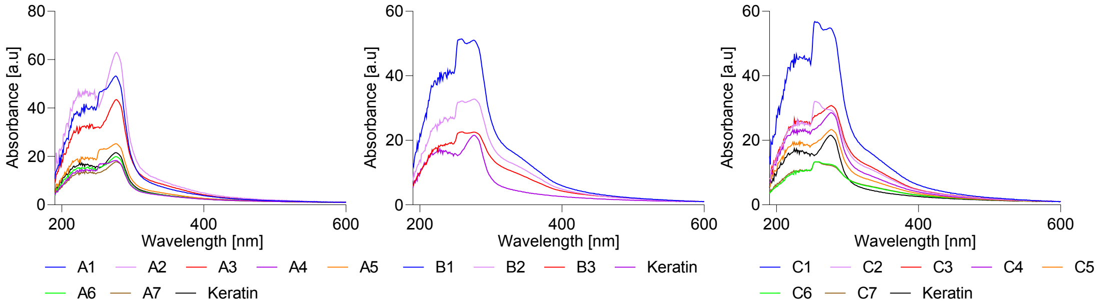

Figure S.1: Normalized UV-VIS spectra of groups A, B, and C, a keratin peak at 276 nm indicated keratin aggregation

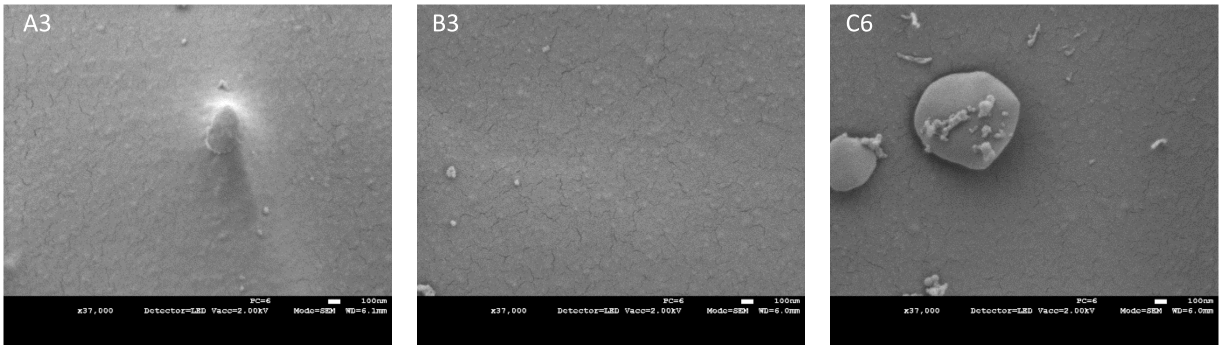

Figure S.2: SEM images of lyophilized KerMel x37,000 magnification shows continuous and smooth morphology compared to keratin.

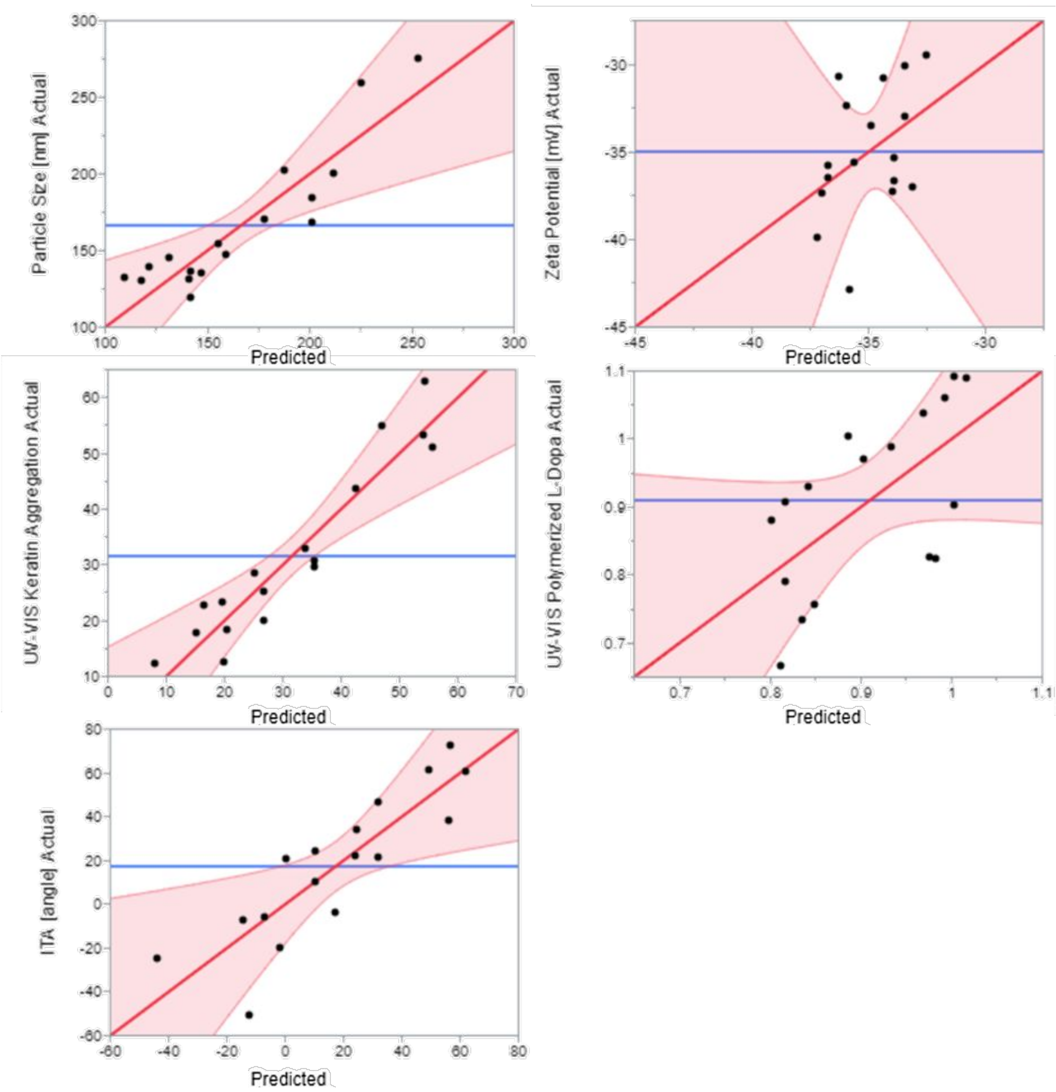

Figure S.3: Actual by prediction plot generated by the DoE of the following responses: particle size, zeta potential, UV-VIS indicated keratin aggregation, ITA, and UV-VIS indicated L-Dopa polymerization.

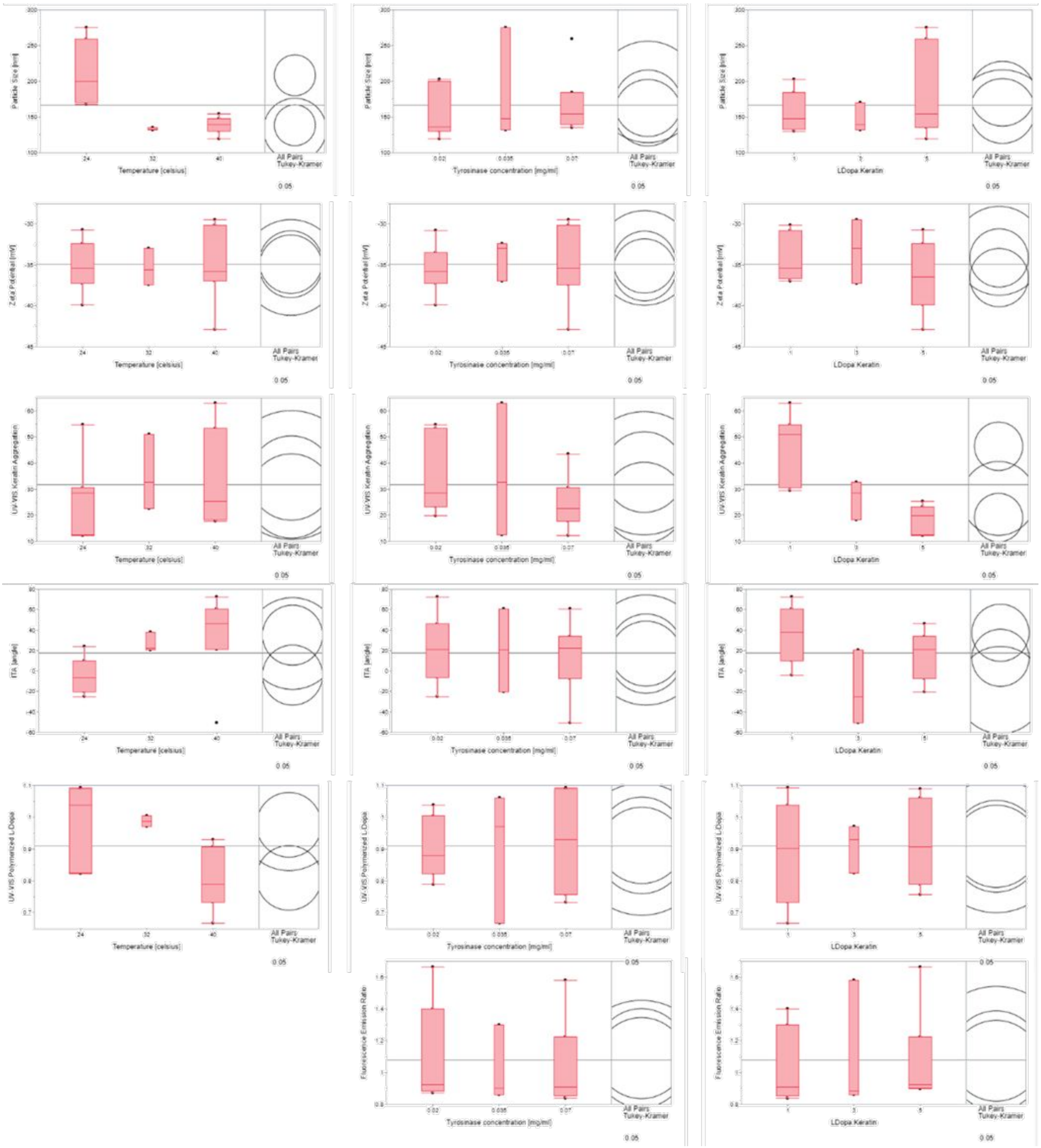

Figure S.4: Tukey-Kramer test of all responses by temperature, TYR concentration, and L-Dopa: keratin ratio, in descending order of particle size, zeta potential, UV-VIS indicated keratin aggregation, ITA, UV-VIS indicated L-Dopa polymerization and fluorescence emission.
